# Within-Epitope Interactions Can Bias CTL Escape Estimation in Early HIV Infection

**DOI:** 10.3389/fimmu.2017.00423

**Published:** 2017-05-01

**Authors:** Victor Garcia, Marcus W. Feldman

**Affiliations:** ^1^Department of Biology, Stanford University, Stanford, CA, USA

**Keywords:** cytotoxic T lymphocytes (CTL), human immunodeficiency virus (HIV), escape, genetic interference, population genetics

## Abstract

As human immunodeficiency virus (HIV) begins to replicate within hosts, immune responses are elicited against it. *Escape* mutations in viral epitopes—immunogenic peptide parts presented on the surface of infected cells—allow HIV to partially evade these responses, and thus rapidly go to fixation. The faster they go to fixation, i.e., the higher their *escape rate*, the larger the selective pressure exerted by the immune system is assumed to be. This relation underpins the rationale for using escapes to assess the strength of immune responses. However, escape rate estimates are often obtained by employing an *aggregation procedure*, where several mutations that affect the same epitope are aggregated into a single, composite epitope mutation. The aggregation procedure thus rests upon the assumption that all within-epitope mutations have indistinguishable effects on immune recognition. In this study, we investigate how violation of this assumption affects escape rate estimates. To this end, we extend a previously developed simulation model of HIV that accounts for mutation, selection, and recombination to include different distributions of fitness effects (DFEs) and inter-mutational genomic distances. We use this discrete time Wright–Fisher based model to simulate early within-host evolution of HIV for DFEs and apply standard estimation methods to infer the escape rates. We then compare true with estimated escape rate values. We also compare escape rate values obtained by applying the aggregation procedure with values estimated without use of that procedure. We find that across the DFEs analyzed, the aggregation procedure alters the detectability of escape mutations: large-effect mutations are overrepresented while small-effect mutations are concealed. The effect of the aggregation procedure is similar to extracting the largest-effect mutation appearing within an epitope. Furthermore, the more pronounced the over-exponential decay of the DFEs, the more severely true escape rates are underestimated. We conclude that the aggregation procedure has two main consequences. On the one hand, it leads to a misrepresentation of the DFE of fixed mutations. On the other hand, it conceals within-epitope interactions that may generate irregularities in mutation frequency trajectories that are thus left unexplained.

## 1 Introduction

*Escape mutations* appear in regions of a viral genome that code for *epitopes*, viral peptides that can elicit immune responses. These responses will frequently consist of cytotoxic T lymphocytes (CTLs) that specifically recognize such epitopes. Escape mutations can emerge during early infection of human immunodeficiency virus (HIV) and commonly go rapidly to fixation (1–6). The emergence and subsequent rise of escape mutations is explained by their net selective advantage (1, 2). A mutation in an epitope-coding region can alter the shape of the epitope, effectively concealing the virus residing within the cell from recognition of the CTL response specific to that epitope. Hence, if no overly deleterious concomitant replicative deficiency is incurred from it, such a mutation allows a strain to replicate at faster rates, which makes it fitter than an unmutated virus strain that is killed at higher rates by CTL.

In recent years, a series of HIV genome analyses from subjects with acute infection have revealed that the majority of escaping epitopes can give rise to multiple escape mutations simultaneously—each determining a unique *escape variant or epitope* (4, 5, 7–9). This phenomenon is termed *epitope shattering* (10). These intra-epitope mutations can display very complex behavior, owed in great part to the differential impact they have on T cell recognition.

The complexity of these dynamical intra-epitope escape patterns induced by T cell pressures is exemplified by the *KK*10 epitope of the *p*24 protein in *Gag*, initially studied by Kelleher et al. (11). Investigations by Schneidewind et al. show that CTL responses specific to the *KK*10 epitope recognize different variants with different efficacy (12). These differential recognition efficiencies are also reported in other studies (13, 14). For *KK*10, the main selected escape variant carries the mutation *R*_264_*K*. Alternative epitope variants with mutations *R*_264_*T*, *R*_264_*Q*, and *R*_264_*G* more effectively abrogate HLA binding, suggesting that they should be preferentially selected. However, the substantial replicative deficiency incurred by these mutations is more difficult to correct by compensatory mutations than for *R*_264_*K*, which is aided by the out-of-epitope mutation *S*_173_*A*. This compensatory mutation restores the fitness of the *R*_264_*K* variant but cannot equally mitigate the replicative fitness costs of the other escape variants. *R*_264_*K* is also associated with the within-epitope precursor mutation *L*_268_*M*, which has only a small replicative fitness cost. Thus, taken together, these findings show that epitope variants may differ in how efficiently they abrogate HLA binding. Furthermore, they strongly suggest that combining different within-epitope mutations into one variant is possible (*R*_264_*K* and *L*_268_*M*) and that strong epistasis may operate in the context of compensatory mutations.

Despite these complications, assessing escape rates in HIV has become a common method to measure CTL killing efficacies (15). Since the growth rate surplus of an escape variant must stem partly from reduced CTL killing, the CTL killing rate is assumed to be at least as large as the *escape rate* of the mutation, the rate at which escape mutations outgrow the unmutated population (2). Thus, time series of escape mutation frequencies obtained from genetic sequencing of blood samples of HIV patients during early infection (2, 4) carry information about CTL killing rates: the faster their rise to fixation, the higher the CTL killing rate. Customarily, in the analysis of these data, the complications arising from epitope shattering phenomena are avoided by *aggregating* the frequencies (i.e., the relative proportions) of all HIV strains that have a mutation in one particular epitope; that is, their frequencies are summed up to give the total frequency of strains that carry a mutation in that epitope (1, 2, 4, 5, 16–18). In the present study, this method will be termed the *aggregation procedure*.

The usefulness of the aggregation procedure rests upon some crucial assumptions. One key assumption posits that mutations that appear within the same epitope are indistinguishable in their effect and may thus be treated as identical. HIV within-host evolution modeling has traditionally adopted this assumption. Following early modeling efforts on escape dynamics (2), a series of deterministic and population-based mathematical models of escape dynamics were published where entire epitopes could either be mutated or not (16, 17, 19–23). Another, later series of stochastic and frequency-based modeling papers also adopted this assumption (18, 20, 24, 25). The rationale behind this notion is that any mutation within any coding part of the epitope will lead to a peptide alteration that fully abrogates HLA binding, and thus completely avoids recognition by the immune system. The evidence on different HLA-binding abrogation effects of escape mutations strongly suggests that this is not always warranted. Nevertheless, how robust standard escape rate estimation techniques are to violation of this assumption remains poorly understood.

To address this issue, we investigated whether the aggregation procedure biases escape rate estimates in a statistically significant manner when within-epitope mutations confer different advantages, and potentially *interfere* (26–28). We studied this question with *in silico* experiments of HIV within-host evolution, using the Wright–Fisher-inspired simulation program developed in Ref. (25). We simulated HIV within-host evolution under different conditions and compared the true input values of selection coefficients of mutations with estimated values, which were calculated by standard estimation procedures, including the aggregation procedure. With this, we extend the investigations of two recent papers that account for within-epitope mutation’s fitness differences to quantify their influence on current escape rate estimates (29, 30).

We extended and further developed the simulation program to incorporate detailed characteristics of HIV. We considered two classes of mutations: one class of mutations in close genomic proximity (within an epitope) and another class at larger genomic distances (between epitopes). We randomly assigned mutations’ positions into different epitopes. We extended the recombination procedure to account explicitly for distances between mutations, affecting how likely they are joined by recombination. Finally, because of their importance to the mode of evolution of a system, we utilized three classes of distributions of fitness effects (DFEs) to run simulations: a fat tailed, an exponential and a short tailed distribution of positive fitness effects (31).

We found that the aggregation procedure tends to conceal mutations of small fitness effect, and thus—relative to an individual-mutation-based estimation approach—overrepresents large-effect mutations. The effect of the aggregation procedure is well approximated by considering only the mutation with the maximum fitness effect within-epitope, neglecting all other mutations. We could not identify any systematic over or underestimation of escape rates by the aggregation procedure relative to individual-mutation-based estimates.

On balance, these results suggest that the widely employed aggregation procedure should be replaced by methods that account for the within-epitope variation of escape mutations.

## 2 Materials and Methods

### 2.1 Simulation Model

We extended a Wright–Fisher model with selection, previously developed to capture features of human immunodeficiency virus (HIV) infections (25), to include two notable features. Here, we briefly describe the core components of the model, which simulates the evolution of different HIV strains present in infected cells only, at discrete time intervals corresponding to one HIV generation. The model also tracks the expansion of virus-infected cells within the host as well as the change of the DNA of the virus residing within them.

HIV strains are assumed to correspond to a sequence of binary loci—a locus corresponds to a codon—which are either in their original state (a zero) or mutated (a one). The wild-type strain, assumed to have ignited the infections, is a strain with only zeros. Zeros mutate into ones at a rate *μ_b_* = 5 × 10^−5^ per locus per replication (see below). No back mutations are considered.

The implementation of replication under selection, as well as recombination, has been described in detail in Ref. (25). Briefly, when selection is acting, each mutation confers a selective advantage. The *fitness w*_**i**_ of a strain **i** is defined as $e^{s_{\text{i}}}$, where **i** = (*i*_1_, …, *i_L_*), ∀*j*: *i_j_* ∈ {0, 1} and *L* is the number of loci (32, 33). A mutation at locus *j* will confer an additional *log-fitness s_j_* to its carrier. Thus, in the absence of epistasis, $w_{i}=\Pi_{j}e^{s_{j}}=\text{exp}\left(\sum_{j}s_{j}\right)$.

The simulation proceeds in two phases. In the first phase, the *neutral phase*, the population undergoes clonal expansion, without selection. On average, one infected cell infects a Poisson-distributed number of new cells, eight on average (34, 35). When the population reaches the upper bound *N* (the *population size*), the simulation proceeds by resampling from the previous generation using a multinomial distribution. The sampling probability *p*_**i**_ of each strain **i** corresponds to the frequency of that strain in the prior generation: *p*_**i**_ = *N*_**i**_/*N*, where *N*_**i**_ is the number of cells infected with strain **i**. After a time delay of *τ_n_* = 14 generations or 28 days, the second phase, the *selection phase*, begins. The population is then resampled from the last generation according to a multinomially distributed random number generator, but with modified sampling probabilities due to selection. The modified probabilities are given by $p_{i,s}=\frac{e^{s_{i}}}{\left\langle e^{s}\right\rangle }p_{i}$, where $\left\langle e^{s}\right\rangle =\sum_{i}\;p_{i}e^{s_{i}}$ (32).

Recombination occurs in only a fraction, *c_i_* = 3%, of infected cells: this is the coinfection rate (36, 37). The template switching rate between strains during reverse transcriptase is *ρ* = 3 × 10^−4^ bp^−1^ (38).

Apart from these core features of the model, we have extended the model to include more biological detail in two ways. First, the model can simulate strains with distinct genomic distances between loci. Second, the selective advantages associated with each locus are drawn from a well-studied exponential-like distribution, which is related to a Gamma distribution. These advantages are determined before the simulation starts and remain fixed over the course of the simulation. These two novel features are described in more detail in the following.

#### 2.1.1 Inter- and Intra-Epitope Mutations

The simulation model can represent mutations that are located at different parts of the genome. To model the inter- and intra-epitope mutations, we chose to include *seven* mutations in each simulation. Two adjacent mutations may be separated on the genome in two ways. Either, a mutation is 10 bp apart from the next one, locating it within the same epitope, or it is 1,000 bp apart, which places it in a different epitope.

For each simulation run, we determined each inter-mutation distance by a random draw, where the probability for a 10 bp distance is 2/7. On average, around two (≈1.7) 10 pb distances will be drawn from six inter-mutation distances. The corresponding mutations will thus be localized in two distinct epitopes, using up around four mutations. The remaining (about three) mutations will constitute their own, single-mutation epitopes, leaving the total number of modeled epitopes at around five (1, 3, 4, 39).

#### 2.1.2 Sampling from Distributions of Fitness Effects

We sampled the selection coefficients for each mutation from a well-studied exponential-like distribution of fitness effects (DFE) (31, 40, 41). The probability density for a mutation to have a selection coefficient *s* > 0, is

(1) $$\rho \left(s\right)=\frac{1}{\sigma }\frac{e^{-{\left(\frac{s}{\sigma }\right)}^{\beta }}}{\Gamma \left(1+\beta^{-1}\right)},$$

where *σ* is analogous to the inverse of a rate parameter in an exponential distribution and *β* is a steepness parameter, indicating over or under-exponential decline. If *β* is one, then *ρ*(*s*) is exactly exponentially distributed.

To sample from the probability density *ρ*(*s*), we show how this distribution is related to a Gamma distribution. The indefinite integral of equation (1) is given by

(2) $$\int \;\rho \left(s\right)\text{ds}=\frac{-\Gamma \left(1∕\beta ,{\left(s∕\sigma \right)}^{\beta }\right)}{\beta \;\Gamma \left(1+1∕\beta \right)}≐F\left(s\right),$$

where $\Gamma \left(a,x\right)≐\int_{x}^{t}\;t^{a-1}e^{-t}\mathrm{dt}$ is the upper incomplete Gamma function and Γ(a)≐Γ(a,0) is the Gamma function. The difference γ(a,x)≐Γ(a)−Γ(a,x) is termed the lower incomplete Gamma function.

We find that requiring the values generated by this density to be positive, the definite integral yielding the cumulative probability distribution of equation (1) is

(3) $$\int_{0}^{s}\;\rho \left(z\right)\mathrm{dz}=F\left(s\right)-F\left(0\right)=\frac{-\Gamma \left(1∕\beta ,{\left(s∕\sigma \right)}^{\beta }\right)}{\beta \;\Gamma \left(1+1∕\beta \right)}+\frac{\Gamma \left(1∕\beta \right)}{\beta \;\Gamma \left(1+1∕\beta \right)}=\frac{\gamma \left(1∕\beta ,{\left(s∕\sigma \right)}^{\beta }\right)}{\Gamma \left(1∕\beta \right)}.$$

The cumulative probability distribution [equation (3)] is therefore a regularized lower incomplete Gamma distribution. This corresponds to the cumulative probability distribution function of a Gamma distribution,

(4) $$\frac{\gamma \left(k,\frac{x}{\theta }\right)}{\Gamma \left(k\right)},$$

where *k* is a shape parameter and *θ* is a scale parameter.

Thus, to sample from the exponential-like distribution, we first defined a Gamma distribution with parameters *k* = 1/*β* and *θ* = *σ^β^*, and then transformed the sample draws *x* from that distribution—by taking the (1/*β*)th power—to obtain the correctly scaled values for the selection coefficient *s*. In the literature, this connection between the exponential-like and the Gamma distribution is typically not mentioned (31, 40, 41).

To model distinct DFE shapes, we ran three sets of simulations with distinct *β*, but equal *σ* = 0.1. The *fat-tailed* distribution (under-exponential decline) is characterized by a *β* = 0.8, the exponential by *β* = 1.0 and the bulky (over-exponential decline) by *β* = 1.4.

#### 2.1.3 Beneficial Mutation Rate

Here, the beneficial mutation rate corresponds to the probability for a within-epitope codon to be altered in a single generation. We assume that the point mutation rate for HIV is *μ* = 2.15 × 10^−5^ bp^−1^ generation^−1^ (42). Within a codon (the length in base pairs is *l_c_* = 3), the chance that a point mutation in the last base pair is not altering the amino-acid coded for, is about *p_w_* = 78%. Thus, the probability not to alter the amino acid per generation is $p_{c}={\left(1-\mu \right)}^{l_{c}}+\mu {\left(1-\mu \right)}^{l_{c}-1}p_{w}$ (probability of no mutation plus probability of altering the last base pair with no consequence). It follows that the probability for a mutation to be altered into an escape codon, that is, for a beneficial mutation to arise is *μ_b_* = (1 − *p_c_*). With these parameter values, we have *μ_b_* ≈ 5 × 10^−5^ bp^−1^ per replication. This value lies between *μ* and the beneficial mutation rate typically assumed for epitopes [10^−4^ per epitope per generation (18, 22)].

### 2.2 Conversion of Escape Rates into Selection Coefficients

Here, we derive a relation between the selection coefficient *s* of a mutation, employed in population genetics theory, and the escape rate *ϵ* of a mutation, employed in virus dynamics studies, following the approach of da Silva (2, 24).

The escape rate of a mutation is the growth rate surplus of a viral strain carrying a beneficial escape mutation relative to some background strain, typically the wild-type strain (2, 17, 20). The proportion of the mutant strain in the entire population follows the time course (2, 17):

(5) $$f\left(t\right)=\frac{f_{0}}{f_{0}+\left(1-f_{0}\right)e^{-\epsilon t}},$$

where *ϵ* is the *escape rate* and *f*_0_ is the initial frequency of the mutant population. Together, *ϵ* and *f*_0_ completely determine *f* (*t*).

To connect *ϵ*, usually measured in units of day^−1^, to the selection coefficient *s*, typically defined in units of generation^−1^, we first define some auxiliary quantities from population genetics. A subpopulation carrying an advantageous mutation is assumed to increase by a growth factor *w* per generation, which Desai and Fisher term *fitness* (28). Here, we use the notation *w_g_* = *w*, where subscript *g* indicates that *w* is measured with respect to generations. The selection coefficient *s_g_* is defined as *log-fitness*, that is, *s_g_* = ln(*w_g_*) (sometimes also confusingly termed fitness). The quantity *w_d_* denotes the same growth factor in units of “per day.” Thus, $w_{d}^{\tau_{g}}=w_{g}$, where *τ_g_* is the generation time in days of the organism in question. In the following, when no subscript is present, we refer to the “per generation” scale.

Following da Silva (24), we now calculate *w_g_* of a strain carrying a single escape mutation. The idea is that the growth factor of the mutant strain must correspond to the ratio of surplus growth rate relative to the wild type (due to reduced killing by CTLs) to the deficit growth rate suffered (due to the fitness cost incurred from the acquisition of an escape mutation). The wild type is killed by CTLs at a fixed rate *k* per day. The mutant strain is often assumed to incur a growth rate reduction of *ψ* per day associated with the acquisition of the escape mutation. Then, the fitness of the mutant strain is *w_d_* = (1 − *ψ*)/(1 − *k*). Thus, $w_{g}=w_{d}^{\tau_{g}}={\left(\left(1-\psi \right)∕\left(1-k\right)\right)}^{\tau_{g}}$. The escape rate of a mutation is defined by *ϵ* ≡ *k* − *ψ*. Here, we ignore fitness costs of escape mutations: *ψ* ≈ 0 day^−1^. Thus, we obtain *ϵ* ≈ *k* and therefore,

(6) $$s\equiv s_{g}\approx \tau_{g}\text{ ln}\;\left(\frac{1}{1-\epsilon }\right)\;.$$

Note that equation (6) with *τ_g_* = 1 day corresponds to an analogous formula given in Ref. (25).

### 2.3 The Aggregation Procedure

In the aggregation procedure, the frequency time course of a multi-mutation epitope is analyzed by regarding all within-epitope mutations as indistinguishable. The frequency of such a multi-mutation epitope will be the sum of the frequencies of all haplotypes that have a mutation within that epitope. Specifically, the frequency *p_e_* of the epitope *e*, will be given by

(7) $$p_{e}=\sum_{\begin{array}{c} i_{k}:k\in \{1,\ldots ,L\}\backslash E\\ i_{j}=1:j\in E \end{array}}\;p_{i_{1},\ldots ,i_{k},\ldots ,i_{j},\ldots ,i_{L}},$$

where *E* is the set of indices of the loci that constitute the epitope *e*. This means that the sum is formed over the frequencies of all haplotypes with a one at a position *j* ∈ *E*. For example, if the second epitope (*e* = 2) has mutations at loci *E* = {2, 3}, and *L* = 3, then,

(8) $$\begin{array}{l} p_{e=2}=\underset{\begin{array}{c} i_{1}\in \left\{0,1\right\}\\ i_{j}=1:j\in \left\{2,3\right\} \end{array}}{\sum }p_{i_{1}i_{2}i_{3}}=\underset{i_{1}\in \left\{0,1\right\}}{\sum }p_{i_{1}10}+p_{i_{1}01}+p_{i_{1}11}=p_{010}+p_{001}+p_{011}+p_{110}+p_{101}+p_{111}. \end{array}$$

The frequency time course of the aggregated mutation frequencies, or aggregates, was analyzed by fitting the logistic-type function [equation (5)] to 1,000 samples of *p_e_*(*t*) taken at different time points. These samples are taken at equal inter-sampling periods, corresponding to sampling every day during the infection. The application of this standard estimation method leads to a single estimate ${\hat{\epsilon }}_{e,\text{aggr}}$ for the aggregate corresponding to epitope *e*. This was subsequently transformed into an estimate of the selection coefficient *s* by means of equation (6): ${\hat{s}}_{e,\text{aggr}}$.

### 2.4 Parameter Values and Their Description

For our simulations we used parameter values as specified in Table 1. If no values are given, they were sampled from density distributions specified above.

**Table 1:** **Parameter values**.

Parameter	Description	Value (if missing: units)
*N*	Population size (18, 25, 43)	10^5^ cells
*L*	Number of loci (3–5)	7
*L_e_*	Number of epitopes (9)	≈5
*μ*	Point mutation rate (42)	2.15 × 10^−5^ bp^−1^ generation^−1^
*μ_b_*	Beneficial mutation rate	5 × 10^−5^ codon^−1^ generation^−1^
*τ_g_*	Generation time of HIV (44–47)	2 days
*τ_n_*	Duration of initial selection-free phase	28 days or 14 generations
*τ_c_*	Simulation cutoff time	1,000 days or 500 generations
*d*	Genomic distance between loci	10, 1,000 bp
*N_r_*	Number of runs per simulation set	2,000
*ρ*	Template switching rate during reverse transcriptase (38)	3 × 10^−4^ bp^−1^
*c_i_*	Coinfection rate (36, 37)	3%
*ϵ*	Escape rate of an escape epitope or mutation	day^−1^
*k*	Killing efficacy of cytotoxic T lymphocytes (CTLs)	day^−1^
*ψ*	Growth detriment imposed by escape mutation (2)	≈0 day^−1^
*β*	Steepness parameter of exponential-like DFE [equation (1)] (28, 31, 41)	0.8, 1, 1.4
*σ*	Inverse rate parameter of exponential-like DFE [equation (1)]	0.1
*f_s_*	Sampling frequency	1 day^−1^
*s*, *s_g_*	Log-fitness or selective advantage per mutation	generation^−1^
*s_d_*	Log-fitness or selective advantage per mutation	day^−1^

## 3 Results

### 3.1 Model Captures Essential Aspects of Early HIV Dynamics

To explore how intra-epitope mutational interactions affect the frequency of mutation trajectories between epitopes, we developed a simulation model for human immunodeficiency virus (HIV) based on previous work (25). The model has been extended to integrate a higher degree of biological realism. Selection acts on several loci simultaneously. Loci can be situated at will in the genome, and therefore the genomic distances between mutations can be modified to produce similar conditions to those observed in early HIV infection. The rates at which mutations at two different loci recombine depend on the genomic distance between them. Mutations can confer different selective advantages, drawn from a distribution of fitness effects (DFE). The fraction of infected cells in which recombination occurs is modeled explicitly, and not by use of an effective recombination rate (25). The population size of the model can be varied.

To analyze how the aggregation procedure is influenced by intra-epitope haplotype dynamics, we adapted our simulation model to mimick conditions observed in empirical studies. Studies of escape dynamics within the first few months of infection usually analyze up to seven CTL-escape epitopes (2–4, 9, 39). A non-negligible fraction of these escape epitopes are aggregations of mutations localized within that epitope, in some instances the majority of epitopes (4).

To capture this feature, we rely on the data presented in Pandit and de Boer (9) to calibrate the fraction of epitopes with multiple mutations simulated: we set up simulations such that up to seven loci can mutate. They can be located within or between epitopes. In the patient analyzed by Pandit and de Boer (5, 9), at the 59th day, four epitopes show escape mutations, out of which two are aggregates of mutations localized within the same epitope. We replicate these conditions by randomly assigning loci into epitopes, as described in *Materials and Methods*.

In the simulations, we used an average selection coefficient per mutation of *s* ≈ 0.1, which corresponds to an average escape rate per mutation of *ϵ* ≈ 0.05, commonly observed in empirical studies (4, 17) (and Supplementary Material therein).

**Figure 1:**
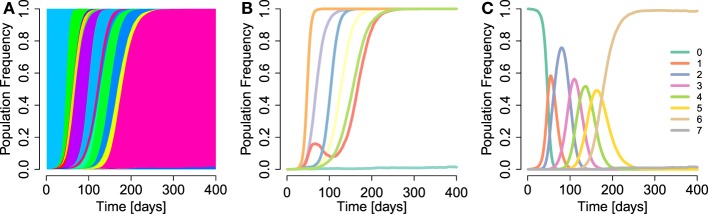
**Example run of simulation model**. **(A)** Haplotype dynamics of a simulation run with parameter values *μ_b_* = 5 × 10^−5^, *N* = 10^5^, *β* = 1, *σ* = 0.1 (specifically, the true selection coefficients sampled were *s*_1_ = 0.0003, *s*_2_ = 0.0742, *s*_3_ = 0.1148, *s*_4_ = 0.0582, *s*_5_ = 0.1139, *s*_6_ = 0.219, and *s*_7_ = 0.0518), *d* = 10, 1,000 bp, and parameters as in Table 1. Each haplotype is attributed a randomly sampled color. **(B)** Frequency trajectories of escape mutations (without aggregating within-epitope mutations). **(C)** Sequential rise and fall of frequencies of haplotypes with *k* mutations, for *k* = 0, …, 7.

Our model appropriately captures the observed timing and fixation patterns of escapes. Figure 1 shows a simulation run of the model. A large variety of haplotypes coexist throughout the simulation, most of them at low frequencies (Figure 1A). Most of the highly advantageous mutations go to fixation before 200 days (Figure 1B). Many of the frequency trajectories of mutations, especially those going to fixation early, appear to follow continuous and regular logistic time courses. As observed in empirical data (4), some trajectories move more erratically: early frequency increases are followed by sudden decreases, which give way to eventual fixation. The fixation trajectories of different mutant frequencies are arranged in such a way that they appear to go to fixation sequentially (2, 17, 22, 39). Our extended model also replicates the phenomenon of *escape rate decrease* (17, 21, 25, 30) (Figure 1B), where subsequent escapes go to fixation after ever longer time spans. We also observe the accrual of beneficial mutations in the population over time (Figure 1C). This accrual is exemplified by the subsequent dominance of *k*-mutants, i.e., haplotypes with *k* mutations. Subsequent waves of ever more mutation-rich haplotypes signify the progression of the population toward higher fitness.

We conclude that the model is able to appropriately describe central features of early within-host evolution and is thus appropriate for investigating the effects of the aggregation procedure on standard estimation techniques.

### 3.2 Simulation Experiments to Assess Aggregation Procedure-Caused Bias

Having established our model’s suitability to capture early HIV within-host evolution, we proceeded to investigate whether the aggregation procedure affects estimates of selection coefficients obtained by standard escape rate estimation techniques.

To this end, we devised two sets of simulation experiments. In our first approach, we took advantage of the fact that each simulation would, by chance, produce a number of epitopes that contain only a single mutation. These *single-mutation epitopes* can be used as a control for the behavior of epitope frequencies that contain multiple mutations—termed *multi-mutation epitopes*—under the aggregation procedure. Within each simulation, each multi- or single-mutation epitope frequency can be analyzed by fitting equation (5) to frequency time-course data, obtaining an estimate $\hat{\varepsilon }$ of the escape rate for each [as done in practice (4, 5, 17)]. The estimate $\hat{\varepsilon }$ is converted into a selection coefficient equivalent $\hat{s}$ by means of the relation (6). Taken together, the estimates from multi-mutation epitopes form a distribution of selection coefficients $\rho_{\text{f},m>1}\left(\hat{s}\right)$, where the subscript f denotes that only fixed mutations (>95% frequency) are analyzed and *m* denotes the number of mutations of the epitope. The single-mutation epitopes give rise to an analogous distribution $\rho_{f,m=1}\left(\hat{s}\right)$, which serves as benchmark.

In a second approach, we compared the distributions of estimated selection coefficients *ρ*_f_($\hat{s}$) obtained by either analyzing all mutations individually within a simulation (without applying the aggregation procedure on any epitope), with distributions obtained employing the aggregation procedure on epitopes $\rho_{\text{f},\text{aggr}}\left(\hat{s}\right)$.

We conducted these simulation experiments in three different regimes, characterized by different shapes of the distribution of fitness effects (DFE). The DFE of HIV is currently unknown, and the aggregation procedure is expected to affect estimates differently depending on the characteristics of the DFE in question. We chose to use a family of DFEs investigated in other studies (28, 31, 41) (see equation (3)). The advantage of this exponential-like DFE is that it can capture different types of decays of density distributions as selection coefficients increase. The characteristics of the decay are largely determined by the steepness parameter *β*.

If *β* < 1, the DFE decays over-exponentially with higher log-fitness *s*. This fat-tailed distribution is known to be associated with *clonal interference* effects. Due to their abundance relative to an exponential decay pattern, small-effect mutations appear frequently, but are likely to be outcompeted by occasional large-effect mutations emerging from the distribution’s fat tail (27).

If *β* = 1, the DFE is an exponential distribution, which has been studied extensively in evolution (27, 41, 48). Under *β* = 1, the simulations should retain signatures of both *β* < 1 and *β* > 1 DFEs.

Distributions with *β* > 1 are bulkier than exponential ones, giving rise to a phenomenon termed *multiple mutations interference* (31) or MMI regime. Under MMI, small-effect mutations are very common, whereas large-effect mutations are extremely rare: lineages that carry advantageous mutations are constantly in competition with newly formed lineages that have acquired different beneficial mutations.

#### 3.2.1 Multi-Mutation Escapes Compared to Single-Mutation Escapes

To explore whether the aggregation procedure causes a bias in the estimation of selection coefficients, we compared the true selection coefficients used to run simulations with the selection coefficients inferred by fitting a logistic-like function to epitope frequency time courses, for both multi- and single-mutation epitopes.

We denote the true maximum within-epitope selection coefficient by $s_{e,\text{max}}=\underset{E}{\text{max}}\;\left\{s_{j_{1}},\ldots ,s_{j_{m}}\right\}$, where *E* = {*j*_1_, …, *j_m_*} is the set of all indices of loci that are localized within the epitope *e* and *m* is the number of loci within epitope *e*. The estimated selection coefficient of a multi-mutation epitope is denoted by ${\hat{s}}_{e,\text{aggr}}$ (see *Materials and Methods*). Note that for a single-mutation epitope ${\hat{s}}_{e,\text{aggr}}={\hat{s}}_{e}$, since *m* = 1.

**Figure 2:**
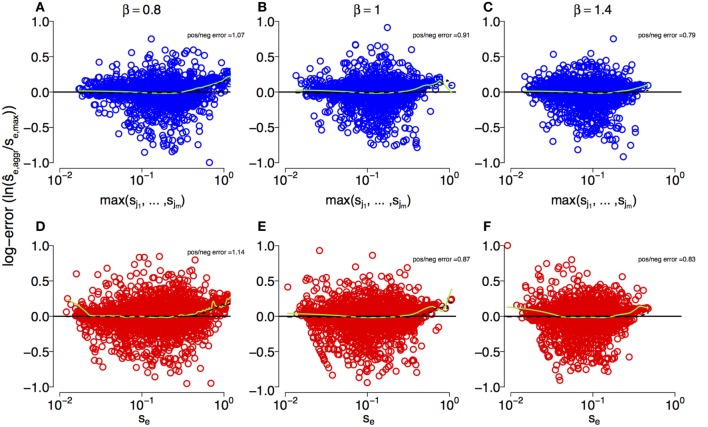
**Log-error of estimates of selection coefficients across different DFEs for multi-mutation and single-mutation epitopes**. **(A–C)** Maximum true within-epitope selection coefficient *s_e_*_,max_ versus the log-error of the estimated selection coefficient ${\hat{\text{s}}}_{\text{e},\text{aggr}}$ in multi-mutation epitopes when applying the aggregation procedure for *β* = 0.8, 1, and 1.4, respectively. The log-error is defined as $\text{ln}\left(\;\right.\frac{{\hat{\text{s}}}_{\text{e},\text{aggr}}}{{\text{s}}_{\text{e},\text{max}}})\;$. **(D–F)** True selection coefficient values *s_e_* versus the log-error of their estimates in single-mutation epitopes. The inset “pos/neg error” is the ratio of the sum of positive log-errors to the sum of negative log-errors. The green lines are a smoothing spline [*smooth.spline* function in the *stats* R-package (49)]. The parameters of the simulation are specified in Table 1.

Figure 2 shows that the estimates ${\hat{s}}_{e,\text{aggr}}$ can deviate substantially from the true simulation input for both multi- and single-mutation epitopes across DFEs. However, we observe that our estimation techniques crudely capture the characteristics of escapes across large spans of *s* values (about three orders of magnitude). This is corroborated by statistical testing: in both multi- and single-mutation epitopes, the distribution of estimated selection coefficient values $\rho_{\text{f}}\left({\hat{s}}_{e,\text{aggr}}\right)$ does not differ significantly from the distribution of simulation input values *ρ*_f_(*s*_*e*,max_) (two-sample Kolmogorov–Smirnov test; see Figure S1 in Supplementary Material). Thus, the estimation methods do not fundamentally alter the characteristics of the distribution of input values. Furthermore, Figure 2 suggests that the effect of the aggregation procedure is well approximated across all DFEs by taking the maximum selection coefficient among within-epitope mutations.

The estimation techniques deliver slightly biased results. To assess bias, we use the sum of the log-error of all overestimates divided by the respective sum of the log-error of all underestimates as a bias statistic (“pos/neg error” in Figure 2). For *β* < 1, this statistic is larger than one, indicating overestimation bias. However, when *β* ≥ 1, true selection coefficient values tend to be underestimated. This effect is produced by the bulk of the estimates, which are centered around the mean of the generating distributions at *s* ≈ 0.1, and is shown by the negative smoothing spline values at that mean in Figure 2. We also observe that toward the front and the rear of the distributions of *s_e_*_,max_ values, overestimates are more common. This is likely to originate from the erroneous conversion of escape rate estimates ${\hat{\epsilon }}_{e,\text{aggr}}$ to ${\hat{s}}_{e,\text{aggr}}$ by means of equation (6), which breaks down for large *ϵ*.

**Figure 3:**
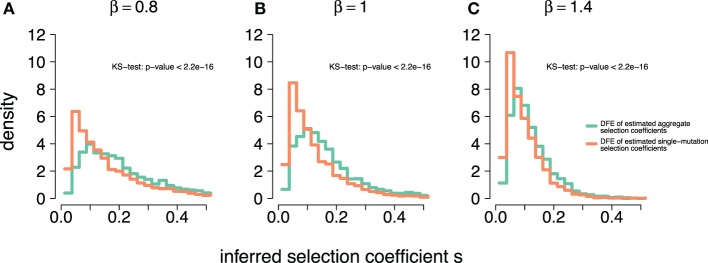
**Aggregation procedure modifies density distribution of inferred selection coefficients of epitopes across DFEs**. **(A–C)** The density distributions of inferred selection coefficients for epitopes containing a single mutation, $\rho_{\text{f},\text{m}=1}\left(\hat{\text{s}}\right)$ (orange line) and epitopes with multiple mutations, $\rho_{\text{f},\text{m}>1}\left(\hat{\text{s}}\right)$ (light green line) for DFEs (as defined in equation (3)) with *β* = 0.8, 1, and 1.4. The parameters for the simulations are specified in Table 1. The inset text shows the p-values of the two-sample Kolmogorov–Smirnov test.

To further investigate the effect of the aggregation procedure with respect to standard estimates, we compared the density distribution of inferred selection coefficients from single-mutation epitopes $\rho_{\text{f},m=1}\left(\hat{s}\right)$ with the distribution from multi-mutation epitopes $\rho_{\text{f},m>1}\left(\hat{s}\right)$. Figure 3 shows both distributions for different DFEs. For all *β*, we find that at small $\hat{s}$, $\rho_{\text{f},m>1}\left(\hat{s}\right)<\rho_{f,m=1}\left(\hat{s}\right)$. However, this relation reverses as $\hat{s}$ becomes larger, leading to $\rho_{\text{f},m>1}\left(\hat{s}\right)>\rho_{\text{f},m=1}\left(\hat{s}\right)$. The aggregation procedure significantly modifies the density distribution of the inferred selection coefficients compared to unaggregated ones (see Kolmogorov–Smirnov tests in Figure 3). Thus, the aggregation procedure reduces the detectability of small-effect mutations, masking them, and overrepresents large-effect mutations. This also explains why the aggregation procedure is well approximated by the maximum function in the comparison in Figure 2. On average, large-effect mutations within an epitope spread first, and conceal the presence of more frequent, small-effect mutations within the same epitope.

#### 3.2.2 Individual-Mutation Analysis Compared to Aggregation Procedure

Since all epitopes to which the aggregation procedure was applied also contained several closely localized mutations, it is unclear whether the observed effects may stem primarily from the aggregation, or alternatively from the clustering of mutations. Because closely clustered mutations are more tightly linked to one another than mutations residing on different epitopes, the effects of interference are likely to be more pronounced within-epitope. Thus, to corroborate previous results, it is necessary to also carry out the analysis on mutations individually. This should be done employing the same standard estimation methods, but in the absence of the aggregation procedure; that is, regardless of the mutation’s relative position in the genome. These individual-mutation-based estimates need to be compared to estimates obtained by applying the aggregation procedure.

**Figure 4:**
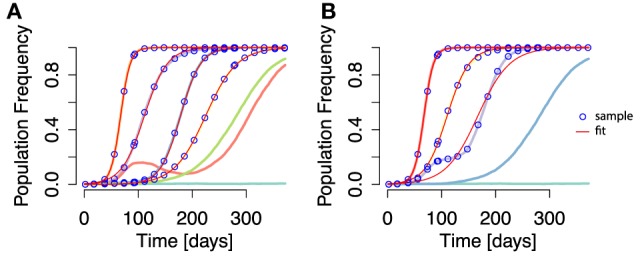
**Time courses of individual mutation frequencies and frequency time courses of epitopes when applying aggregation procedure (both shown in lines of randomly chosen colors)**. **(A)** Individual mutation based analysis: each frequency time course of a mutation, irrespective of the mutation’s position in the genome, is analyzed and the escape rate estimated. **(B)** The aggregation procedure is applied, and mutations within the same epitope are collapsed into an aggregate epitope frequency. There are thus fewer frequency time course lines than in **(A)**. Here, epitope frequency time courses are used for escape rate estimation. The blue points are the sampled frequencies. The thin red lines are the fit of equation (5) to the sampled frequencies. In some cases, the fit line appears on top of both mutation and aggregate epitope frequency time courses. Simulation parameters are specified in Table 1.

To this end, we devised a second set of simulation experiments, where we compared (i) selection coefficient estimates from each individual fixed mutation within each simulation with (ii) the estimates of selection coefficients of fixed epitopes under the aggregation procedure. More specifically, for each simulation we performed two types of analysis: (i) one in which the escape rate of each mutation that goes to fixation (irrespective of its position in the genome) is inferred by fitting the logistic-type function [equation (5)] (see Figure 4A) and (ii) one in which mutations residing within the same epitope are aggregated, and the logistic-type function is applied to both aggregated and non-aggregated epitopes (see Figure 4B).

As in the first approach, estimates stemming from both perspectives are transformed into selection coefficient equivalents and may be compared in terms of their distributions. All individual-mutation-based escapes under (i) across simulations make up a list of selection coefficient estimates. Taken together, these form a distribution ${\hat{\rho }}_{\text{f}}\left(\hat{s}\right)$. Analogously, (ii) leads to a list of selection coefficient estimates from multi-mutation epitopes as well as single-mutation epitopes. These form the distribution ${\hat{\rho }}_{\text{f},\text{aggr}}\left(\hat{s}\right)$.

**Figure 5:**
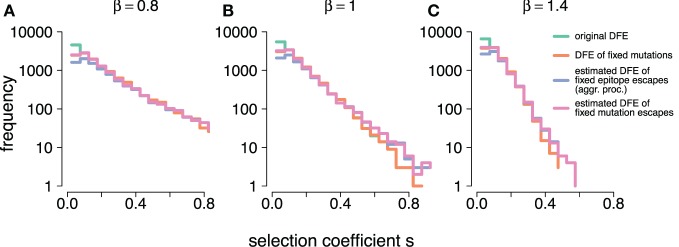
**(A–C)** show the frequency distributions of the true selection coefficients, $L\cdot N_{r}\cdot \rho (s)$ (original DFE), the selection coefficients of mutations that went to fixation, ${\text{N}}_{\text{f},\text{s}}\cdot \rho_{\text{f}}\left(\text{s}\right)$ (DFE of fixed mutations), the estimates obtained under the aggregation procedure, ${\text{N}}_{\text{f},\text{aggr}}\cdot {\hat{\rho }}_{\text{f},\text{aggr}}\left(\hat{\text{s}}\right)$ (estimated DFE of fixed epitope escapes (aggr. proc.)), and the individual-mutation based estimates, ${\text{N}}_{\text{f}}\cdot {\hat{\rho }}_{\text{f}}\left(\hat{\text{s}}\right)$ (estimated DFE of fixed mutations escapes), for all simulated DFEs (β=0.8,1 and 1.4, respectively). At small *s*, ${\text{N}}_{\text{f},\text{s}}\cdot \rho_{\text{f}}\left(\text{s}\right)$ and ${\text{N}}_{\text{f}}\cdot {\hat{\rho }}_{\text{f}}\left(\hat{\text{s}}\right)$ overlap. At large *s*, $L\cdot N_{r}\cdot \rho (s)$, and ${\text{N}}_{\text{f},\text{s}}\cdot \rho_{\text{f}}\left(\text{s}\right)$ overlap. Simulation parameters are specified in Table 1.

Figure 5 shows how the frequency distributions of estimated selection coefficients, $\hat{s}$, with and without the aggregation procedure compare to one another and to the true selection coefficients, *s*, and selection coefficients of fixed mutations across all DFEs. We observe that the frequency distribution of all simulation-generated true selection coefficients *L* ⋅ *N_r_* ⋅ *ρ*(*s*) (see Table 1 for values of *L* and *N_r_*) is largely equivalent to the frequency distribution of true selection coefficients of mutations that went to fixation, *N*_f,_*_s_* ⋅ *ρ*_f_(*s*), where *N*_f,_*_s_* is the total count of mutations that went to fixation. They differ only at small selection coefficient values. This is due to the simulation cutoff time *τ_c_*, which leads to small-effect mutations not reaching the 95% threshold in time to be considered fixed.

These two frequency distributions of true supplied and true fixed selection coefficients show fundamental differences from models analyzed in other studies, for example (31). In Ref. (31), small-effect mutations are lost either by drift or being outcompeted by a constant supply of large-effect mutations. Because large-effect mutations are interspersed across simulation time, on average they may affect the trajectories of small-effect mutations at any time point. In our simulation framework, however, the supply of beneficial mutations is limited (*L* = 7). Thus, large-effect mutations are likely to have established early in the dynamics, and their supply is exhausted after all have gone to fixation. This leaves the remaining small-effect mutations free from extinguishing competition, which allows them to go to fixation unimpaired.

Figure 5 also confirms the insights from our previous analysis. At large *s*, the frequency distribution of estimated selection coefficients both with and without the aggregation procedure ($N_{\text{f},\text{aggr}}\;\cdot \;{\hat{\rho }}_{\text{f},\text{aggr}}\left(\hat{s}\right)$ and $N_{\text{f}}\;\cdot \;{\hat{\rho }}_{\text{f}}\left(\hat{s}\right)$, respectively, where *N*_f,aggr_ and *N*_f_ are the counts of mutations that went to fixation under each respective procedure), surpass the true supply of mutations. This is in line with the observation of a systematic positive bias in the log-errors of the estimates at large *s*. Furthermore, $N_{\text{f},\text{aggr}}\;\cdot \;{\hat{\rho }}_{\text{f},\text{aggr}}\left(\hat{s}\right)$ and $N_{\text{f}}\;\cdot \;{\hat{\rho }}_{\text{f}}\left(\hat{s}\right)$ become very similar, suggesting that large-effect mutations, as identified and estimated under the individual-mutation-based analysis, are equally visible under the aggregation procedure. At $\hat{s}$ values around the mean of the generating DFEs, we observe that $N_{\text{f},s}\;\cdot \;\rho_{\text{f}}\left(s\right)\approx N_{\text{f}}\;\cdot \;{\hat{\rho }}_{\text{f}}\left(\hat{s}\right)$. This suggests that the individual-mutation-based estimates are able to capture most if not all of the supplied mutations with small selection coefficient values. We further observe that with decreasing $\hat{s}$, $N_{\text{f},\text{aggr}}\;\cdot \;{\hat{\rho }}_{\text{f},\text{aggr}}\left(\hat{s}\right)<N_{\text{f}}\;\cdot \;{\hat{\rho }}_{\text{f}}\left(\hat{s}\right)$. Because the aggregation procedure on a simulation run will collapse the mutant frequencies of all within-epitope mutations into a single epitope frequency, there must be fewer selection coefficient estimates under the aggregation than there are loci, since the number of epitopes *L_e_* is typically smaller than the number of potential mutations *L* = 7.

These observations imply that the aggregation procedure leads to an overestimate of large selection coefficients, as well as an underestimate of small values of selection coefficients, consistent with our earlier finding. The distributions thus also confirm that the aggregation procedure will mask low-effect mutations, and overrepresent large-effect mutations.

### 3.3 Aggregation Procedure Can Conceal Strong Within-Epitope Sweeps That Affect Other Epitopes

**Figure 6:**
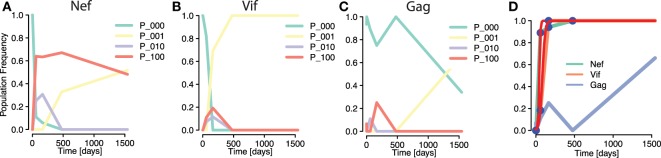
**Masking of strong within-epitope mutation interactions through aggregation procedure in escapes from one patient Ref. (5) [epitope variant reconstruction in Ref. (9)]**. **(A–C)** Frequencies of distinct epitope variants within HIV epitopes *Nef A24-RW8*, *Vif B38-WI9*, and *Gag A01-GY9*, respectively. Three different mutations were measured within each epitope. The epitope variants are denoted by *P*_*i*_1_*i*_2_*i*_3_, where *i_j_* = 1 denotes a mutation in the *j*th considered locus within the epitope (*P*_000 is the wild type). All within-epitope variant dynamics show intense interference effects. **(D)** The fit of a logistic escape model (red line) to the sample points of *aggregated* escape mutant frequencies within one patient. Multiple escape mutations appear within the epitopes of the genes *Nef* and *Vif* and tGag, whose frequencies are summed into one aggregate escape mutant frequency per epitope [Figure 6C in Ref. (22)]. The sampling times are 0, 3, 59, 165, 476, and 1,543 days after infection was determined. Despite the strong within-epitope interference in *Nef* and *Vif*, the trajectories of the aggregates appear to be regular. The trajectory of the *Gag* is irregular due to the influence of a within-epitope sweep in *Vif*, as revealed by the analysis of Pandit and de Boer (9).

To compare the simulations with data, we investigated an instance of early HIV infection in a patient for which haplotype sequences were reconstructed. Henn et al. (5) performed whole genome deep sequencing on samples from the patient using 454 pyrosequencing techniques. These data were reconstructed to HIV strains by Pandit and de Boer (9), allowing frequencies of within-epitope escape variants—or *strains*—to be tracked over time. Pandit and de Boer identified interference effects among mutations within the same epitope (see Figures 6A,B), but also among mutations between different epitopes (Figures 6B,C). Crucially, differences in selective advantages of mutations in the same epitopes lead to within-epitope selective *sweeps*, reductions of genetic diversity by the fast establishment and subsequent fixation of a mutation (Figure 6B). The haplotype or strain frequencies revealed that these sweeps affected frequencies of mutations in other epitopes (9). In fact, Figures 6B,C show how a within-epitope sweep in one epitope (*Vif B38-WI9*) causes the frequencies of some variants of another epitope (*Gag A01-GY9*) to vanish, and with it, the total frequency of all variants of that epitope (Figure 6D). However, the effects of the frequency decline of these epitope variants were concealed by the aggregation procedure. This instance shows how focusing only on the aggregate frequencies can mask the real causes of observed frequency fluctuations in epitopes.

The aggregation procedure may thus lead to an altered perception of escapes in two ways: on the one hand, it obscures the within-epitope causes of the delayed fixation of a different epitope (a between-epitope interaction). On the other hand, it misrepresents between-epitope interactions, leaving the irregularities in mutation frequency trajectories unexplained.

## 4 Discussion

In this study, we analyzed the effects of the aggregation procedure on currently employed standard techniques for escape rate estimation. To this end, we further extended an early-infection model of within-host HIV evolution, based on a Wright–Fisher framework employed in our previous work (25). The new features of our model incorporate some biological details of HIV infection that were previously neglected: (i) the relative location of the sites of escape mutations, which can either be located very closely together within epitopes or far apart in different epitopes in the genome (4, 10), (ii) the rate at which mutations arise and recombine given their relative genomic distances, and (iii), the fitness attribution to mutations according to three distinct distributions of fitness effects, implying that fitness effects of within-epitope mutations differ because they induce different CTL-recognition losses (12).

We adopted two independent approaches to assess how escape rate estimates are affected by the aggregation procedure: (a) by comparing the estimate distributions for within-epitope aggregates of mutations with estimate distributions from single-mutation epitopes and (b) by comparing estimated fitness effect distributions obtained by applying the aggregation procedure to all epitope-coding regions individually with estimated distributions obtained by analyzing all mutations individually.

We found that in both approaches and for all examined DFEs the aggregation procedure tends to conceal escapes of mutations with small fitnesses while overrepresenting large-fitness mutations. The effect of the aggregation procedure is well approximated by selecting the mutation with largest fitness occurring within an epitope. This is due to the tendency of fitter mutations to go to fixation earlier than the less fit mutations. In such a scenario of fitness-ordered escapes, the application of the aggregation procedure results in the detection of the first within-epitope mutation that goes to fixation, which also tends to be the mutation with highest fitness.

Irrespective of the application of the aggregation procedure, the estimation techniques employed here appear to underestimate true selection advantages at the DFE’s mean, where the bulk of the selective advantages of the generated mutations reside: around *ϵ* ≈ 0.05 [day^−1^], or equivalently, *s* ≈ 0.1 [generation^−1^]. Conversely, the estimation methods tend to systematically overestimate the true value for large *s*, due to the break down of the relation that converts inferred escape rates *ϵ* to selection coefficients *s*.

Despite the incorporation of further biological detail and its ability to capture some important aspects of early HIV infection, by necessity our model must rely on some simplifications of the very complex immunological interactions in attempting to mimic HIV within-host evolution. Mismatches between model behavior and data may previously have been plausibly attributed to some neglected facets of HIV’s biology, such as recombination or variation in fitness effects. Thus, their incorporation allows us to reassess whether these mismatches stem from more central assumptions inherited from previous models.

Correspondingly, one of the caveats of this study lies in the assumption of a finite supply of beneficial mutations. This assumption is based on the observation that most early adaptation in HIV occurs at a limited number of loci subject to strong selection (50), usually located in the *Env* and *Nef* genes (3, 51, 52). The relative strength of the CTL responses, as well as their breadth, is hypothesized to determine immune escape (53, 51). Because up to eight epitope-specific CTL responses may emerge during acute infection (3), modeling a similar number of sites is assumed to sufficiently reflect early adaptive dynamics. This assumption of a finite mutation supply, typically used when no within-epitope variation is posited, can alter in important ways the evolutionary dynamics relative to a supply-rich scenario, where the vast majority of epitopes exhibit shattering. That is, elimination of small-effect mutations by rare but recurrent large-effect mutations is suppressed. Thus, all mutations, irrespective of their fitness effect, eventually go to fixation if enough time elapses. Accordingly, patterns observed in empirical studies, where some beneficial mutations do not reemerge after having been outcompeted by fitter ones, are only temporary in our simulation experiments.

In patient data, the simultaneous emergence of several within-epitope mutations—each corresponding to a variant—is sometimes followed by the fixation of a single mutation [for example, Figure 3B in Ref. (4), see also Ref. (8, 30)]. Patient data suggest that mutations within epitopes may not necessarily be beneficial when appearing in combination. In fact, it has been suggested that these early epitope variants are often mutually exclusive (10). This may be due to strong epistatic effects between either within-epitope mutations themselves, or between compensatory mutations and within-epitope mutations. Here, we have neglected the effect of such epistasis. Alternatively, the transient nature of within-epitope genetic variation may have been imperfectly replicated in our simulations due to the aforementioned scarce beneficial mutation supply.

In this study, the replicative deficit—termed fitness cost—and the fitness gain due to reduced CTL recognition incurred from an escape mutation are combined into a single effective selection coefficient. With this, we implicitly assume that the advantage from partial CTL-recognition loss induced by an escape mutation may vary from mutation to mutation, as suggested by experimental evidence (12, 30). We neglect the effect of compensatory mutations due to the assumptions that compensatory mutations arise and go to fixation rapidly, that high-cost mutations are rare (54) and that their fitness effects are small relative to CTL pressures (2). By attributing a constant fitness value to each mutation, we also neglect the effect of varying CTL numbers—a key problem in HIV modeling (29).

How the internal environment of the human host shapes the availability of beneficial mutations is largely unknown. It remains unclear what determines the immunodominance hierarchy of immune responses, although the host’s HLA profile must play an important role (52). How the relative strength of these responses translates into selective pressures—and thus DFEs—remains a topic of investigation (24, 25). du Plessis et al. (55) have computed the DFE of HIV by means of a model that predicts HIV strain’s fitness based on previous work by Hinkley et al. (56). They found that a substantial proportion of the randomly sampled genetic neighborhood of a reference strain contained beneficial mutations but did not statistically analyze the shape of the resulting DFE. Given this lack of information, here, we explored a limited variety of DFEs thought to assume biologically plausible shapes (31, 41). We restricted ourselves to varying only one shape parameter of that exponential-like DFE, *β*.

Another caveat lies in the assumption of a constant population size *N* after an early period of population expansion. The shortcomings associated with this assumption have already been discussed in depth in Garcia et al. (25). Briefly, a constant population size may misrepresent fluctuations that arise during early HIV infection, such as a spike in viral load around 3 weeks after infection. However, we are more focused on the number of cells within which HIV replicates, because this better reflects the genetic composition of the viral population. Several studies of HIV’s genetics have shown that models with a constant population size can replicate several essential features of HIV’s genetic diversification process (18, 20, 43).

Also, the prevalence of multi-mutation epitopes might have been too low in simulations. Our calibration of this prevalence was based on the study of Pandit and de Boer (9), which discusses data from a single patient previously analyzed in Ref. (5). However, the three patients in Ref. (4) almost exclusively show epitopes with multiple mutations. The choice to use the Pandit and de Boer study as calibration reference was motivated by seeking a more direct way to compare simulation outcomes that aggregate within-epitope mutations with individual analysis, while keeping computational times reasonably low.

The idea that the aggregation procedure might affect the reliability of estimation methods for escape rates is connected to the notion that trajectories of mutations affect one another. The non-independent behavior of tightly linked mutations as they go to fixation is commonly associated with genetic *interference*: because advantageous mutations cannot combine into the same genetic background, a competitive state arises between them, in which a frequency gain of one mutation implies a reduction in frequency of other mutations. Mutations that interfere with one another in this way also delay each other’s fixation, creating a mismatch between the theoretical escape rates when each evolves independently and observed escape rates.

The importance of interference in HIV early infection remains unclear (25): on the one hand, sequential accrual of escape mutations appears consistent with some patient data (39) and the low estimated effective population sizes combined with decreasing immune pressures across CTL clones (24). This explanation of the viral genetics during early within-host evolution does not necessitate interference. On the other hand, haplotype reconstruction techniques and single genome amplification data from several patients reveal the coexistence of several viral strains differing at multiple sites (5, 9, 23), which is consistent with clonal interference.

Methods that correct for possible interference effects are needed. The study of Kessinger et al. (18) presents a framework in which these challenges might be addressed in the future. In their analysis of escape mutations, however, some simplifying assumptions were made, such as the sequential acquisition of beneficial mutations, which does not fully account for interference. Furthermore, the aggregation procedure was also applied to some epitopes. In another study, Leviyang has developed escape rate estimation methods for scenarios with multiple within-epitope mutations, but these methods are limited by current HIV sequence data precision (29).

Very few studies have investigated how interference effects manifest themselves when mutations within- and between-epitopes influence one another. A recent paper by Batorsky et al. (30) offers a mechanism for the transient appearance of within-epitope variation as observed in patient data. First, they distinguish between three main dynamical within-epitope escape patterns: a common *sweep* pattern where a single mutation goes to fixation, a *leap-frog* pattern in which a transient epitope variant is eventually outcompeted by another variant and finally, a *nested* pattern, where early escape variants are replaced by variants that incorporate the former variant’s mutations while carrying additional ones. To replicate these patterns, they develop a mathematical model where all mutations are associated with a fitness cost Δ*f* as well as with selective advantage Δ*r* due to evasion from recognition by CTLs. Both Δ*f* and Δ*r* are assumed to be uniformly distributed. Batorsky et al. were able to show that mutations with large Δ*r* and low Δ*f* would naturally appear first in HIV’s within-host evolution, followed by mutations with smaller Δ*r* and larger Δ*f*. This replacement mechanism was consistent with observed features of within-epitope genetic variation, where different haplotypes may coexist for a substantial time period.

Batorsky et al.’s results confirm that within-epitope HIV dynamics, as expressed in *epitope shattering* (10, 29, 57) may not be trivially disentangled from between-epitope dynamics (30). The integration of within- and between-epitope perspectives into a unifying picture requires further work. Accounting for restricted recombination between mutations that may lie close together in the genome or, alternatively, be very distant from each other, adds considerable complexity. Nevertheless, the richness of the phenomena produced by their interplay promises to open up novel means to study early within-host evolution of HIV and how it is shaped by the human immune system.

## Author Contributions

VG and MF designed the work; analyzed and interpreted the data; and wrote the manuscript. VG performed the simulation experiments.

## Conflict of Interest Statement

The authors declare that the research was conducted in the absence of any commercial or financial relationships that could be construed as a potential conflict of interest.

## Supplementary Material

Figure S1 **True versus inferred selection coefficients for multi-mutation and single-mutation epitopes across all DFEs**. **(A–C)** The maximum true within-epitope selection coefficients versus the inferred selection coefficient of their aggregate for *β* = 0.8, 1, and 1.4 in multi-mutation epitopes. **(D–F)** The true selection coefficient values of single-mutation epitopes versus their inferred values. The black diagonal line is where true and estimated values are equal. The gray line is a Theil-Sen estimator regression.
