# Supplementary figures and images for: Within-Epitope Interactions Can Bias CTL Escape Estimation in Early HIV Infection
Source: Front Immunol. 2017 May 1;8:423. doi: 10.3389/fimmu.2017.00423 (PMC5410659; doi:10.3389/fimmu.2017.00423)

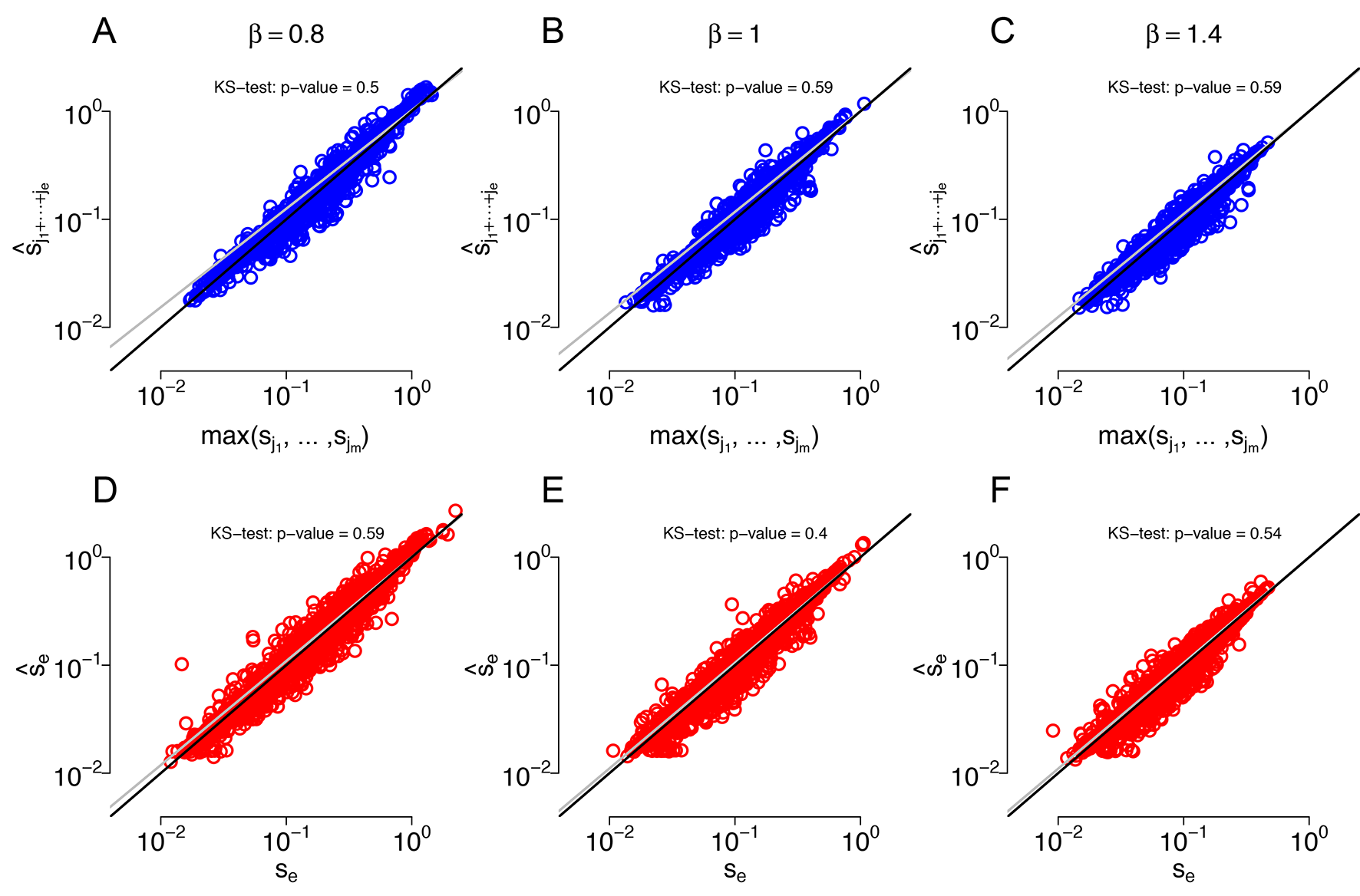

Supplement: Figure S1 — True versus inferred selection coefficients for multi-mutation and single-mutation epitopes across all DFEs. (A–C) The maximum true within-epitope selection coefficients versus the inferred selection coefficient of their aggregate for β = 0.8, 1, and 1.4 in multi-mutation epitopes. (D–F) The true selection coefficient values of single-mutation epitopes versus their inferred values. The black diagonal line is where true and estimated values are equal. The gray line is a Theil-Sen estimator regression. [file image_1.tif]
